# In Vitro Antioxidant Activities of Plant Polyphenol Extracts and Their Combined Effect with Flaxseed on Raw and Cooked Breast Muscle Fatty Acid Content, Lipid Health Indices and Oxidative Stability in Slow-Growing Sasso Chickens

**DOI:** 10.3390/foods12010115

**Published:** 2022-12-26

**Authors:** Desalew Tadesse, Negussie Retta, Mekonnen Girma, Nicholas Ndiwa, Tadelle Dessie, Olivier Hanotte, Paulos Getachew, Dirk Dannenberger, Steffen Maak

**Affiliations:** 1 Department of Animal Production and Welfare, Mekelle University, Mekelle 231, Ethiopia; 2 Center for Food Science and Nutrition, Addis Ababa University, Addis Ababa 1176, Ethiopia; 3 LiveGene, International Livestock Research Institute (ILRI), Addis Ababa 5689, Ethiopia; 4 Research Methods Group, International Livestock Research Institute (ILRI), Nairobi 30709, Kenya; 5 Center for Tropical Livestock Genetics and Health (CTLGH), The Roslin Institute, Edinburgh EH25 9RG, UK; 6 School of Life Sciences, University of Nottingham, Nottingham NG7 2UH, UK; 7 Research Institute for Farm Animal Biology (FBN), 18196 Dummerstorf, Germany

**Keywords:** plant extracts, flaxseed, slow-growing Sasso chickens, *n* − 3 PUFA, lipid oxidative stability

## Abstract

Adding flaxseed was found to decrease oxidative stability in feed and increase the antioxidant needs of chicken. This has also been associated with a decrease in the nutritional value and oxidative stability of meat if sufficient dietary antioxidants are not included. Furthermore, dietary flaxseed has been explored in fast-growing chickens as such studies are limited with slow-growing chickens. Thus, this study aimed to evaluate the effects of feeding plant polyphenol extracts as an antioxidant alongside flaxseed on fatty acid content, oxidative stability, and lipid health indices in breast muscle of slow-growing Sasso T451A dual-purpose chicken. A total of 126 chickens assigned to six groups (seven replicates of three) were fed on NC (control and no antioxidants), FS (75 g flaxseed and no antioxidants), VE8 (75 g flaxseed and 800 mg vitamin E), TS8 (75 g flaxseed and 800 mg *Thymus schimperi*), DA8 (75 g flaxseed and 800 mg *Dodonaea angustifolia*) and CD8 (75 g flaxseed and 800 mg *Curcuma domestica*) extract per kg diet. Feeding on CD8 and VE8 in raw and TS8, CD8 and VE8 diets in cooked breast muscle increased (*p* < 0.05) the C22:6*n* − 3 (DHA) and C20:5*n* − 3 (EPA) contents compared to the FS diet. Feeding FS increased (*p* < 0.05) the malondialdehyde (MDA) content in breast muscle, whereas TS8 in cooked and raw and CD8 and DA8 diets in raw breast muscle decreased it (*p* < 0.05). No added benefit was observed in feeding VE8 over plant extracts in terms of improving fatty acid composition and lipid health indices and reducing lipid oxidation in breast meat.

## 1. Introduction

Most poultry industries’ primary focus is on increasing meat and egg production. However, it should also focus on the production of healthy eggs and meat for improved consumer health [1]. One important nutritional and health concern that needs to be addressed in chicken egg and meat is the ratio of omega-6 to 3 fatty acids. Commercial chicken meat is identified to be deficient in omega-3, but rich in omega-6 fatty acids. However, a high intake of omega-6 fatty acids is correlated with negative health impacts [2]. Despite having several health benefits in preventing cardiovascular and degenerative diseases [3], there is a gross deficiency of omega-3 fatty acids, particularly in eicosapentaenoic acid (EPA) and docosahexaenoic acid (DHA), globally [4]. Marine fish and microalgae are primary sources of *n* − 3 long-chain polyunsaturated fatty acids (*n* − 3 LC PUFAs) including EPA and DHA. However, the marine ecosystem is under constant pressure raising the issue of long-term sustainability of the sector [5]. Thus, it is vital to explore other suitable *n* − 3 LC-PUFA sources [6]. Chicken meat is widely consumed worldwide [7] and is more accessible than other meat types to poor households in low-income countries [8]. Hence, enriched chicken meat represents a viable vehicle to enhance the human intake of *n* − 3 LC-PUFAs.

Flaxseed/linseed (*Linum usitatissimum* L.) has been included in chicken diets as an alpha-linolenic acid (ALA) precursor to enrich egg and meat with *n* − 3 LC- PUFA [9,10], and there is an increased interest to add more flaxseed in the chicken diet [11,12]. However, there are still challenges affecting the full use of flaxseed in chicken diets. Firstly, chickens have limited efficiency in converting dietary ALA precursor to EPA and DHA [13,14]. Secondly, the higher inclusion of dietary fat in chickens induces oxidative stress in vivo [15], leading to lipid peroxidation. Thus, ways to improve the conversion efficiency of dietary ALA to EPA and DHA, enhance oxidative stability and improve lipid health indices in the resulting meat needs to be carefully investigated. To prevent the problem of lipid peroxidation, synthetic antioxidants have been applied, but their residues in the meat carcasses have raised health concerns due to their carcinogenic properties [16,17]. Several studies have suggested the inclusion of natural antioxidants in chicken diets as a solution to the problem of lipid peroxidation [18,19,20].

Plants rich in polyphenols have a significant capacity to counteract free radicals and eliminate peroxidation reactions [21]. However, studies on the effects of plant polyphenol extracts as dietary antioxidants in animals are few [22]. Moreover, there is limited research into the potential effects of adding plant extract antioxidants in flaxseed-supplemented chicken diets on fatty acid compositions, lipid stability, and lipid health indices. Furthermore, studies are needed to evaluate the effectiveness of various plant dietary antioxidants in raw and cooked meat to offer multiple sources of natural antioxidants. A recent study showed that the inclusion of dietary flaxseed with turmeric rhizome powder (TRP) as a polyphenol antioxidant has improved the *n* − 3 LC-PUFA content and oxidative stability of chicken meat [23]. Moreover, combined flaxseed and green tea polyphenols supplementation enhanced the activity of lipid metabolizing enzymes [24].

The diversity of medicinal plants available in Ethiopia could offer the opportunity to extract effective antioxidants to inhibit oxidative stress [25]. *Dodonaea angustifolia* (*D. angustifolia*) has been utilized to treat lymphatic swelling and burn healing [26] and was found to be an effective antioxidant against peroxidation in vitro [27]. *Thymus schimperi* (*T*. *schimperi*) has been commonly used as a traditional herbal tea drink. It is believed to treat cough and liver diseases [28], and to be good for diabetic patients [29]. Additionally, *Curcuma domestica* (*C. domestica*), which has antioxidant properties, is a common ingredient in every Ethiopian kitchen for the preparation of traditional sauces and had several health benefits [30,31,32].

Up to now, the effect of the inclusion of flaxseed as an *n* − 3 PUFA source in chicken diets has been tested widely in fast-growing commercial meat [21,33,34] and egg layer types [35,36]. Studies in slow-growing dual-purpose chickens are limited [37,38]. In this study, it was hypothesized that the use of plant polyphenol extracts (PPEs) of *D. angustifolia*, *T. schimperi*, and *C. domestica* extracts along with dietary flaxseed could improve the muscle fatty acid composition, oxidative stability, and lipid health indices of SassoT451A slow-growing dual-purpose chicken. More specifically, the study aimed to determine the effects of the supplementation of *T. schimperi, D. angustifolia,* and *C. domestica* extracts as polyphenol antioxidants and a higher dose of vitamin E, along with flaxseed as an ALA precursor in the diets of slow-growing Sasso chicken on fatty acid content, oxidative stability, and lipid health indices in raw and cooked breast meat.

## 2. Materials and Methods 

### 2.1. Experimental Design, Diets, and Birds

The current study protocols were approved by the Institutional Animal Care and Use Committee (IACUC2019-17.2) of the International Livestock Research Institute (ILRI), following the Sasso dual-purpose chicken management guide obtained from Hendrix Genetics (FGS, 2018, p. 7–11) [39]. At 35 weeks, 126 Sasso T451A chickens were randomly assigned using incomplete randomized block design into six different dietary treatments with 3 birds per pen (21 birds per treatment, 7 replicates of 3 birds). A soybean corn-based diet was formulated to meet the nutrient requirements of Sasso T451A as per [39], with sunflower and soybean meals added to the control diet to balance the fat added from flaxseeds in other diets. The diets were mixed in a rotating drum mixer for 30 min, with plant extracts and vitamin E (α-tocopherol acetate) incorporated into a small amount of wheat bran before inclusion into the main ingredients to ensure precise dispersal throughout the whole feed. 

The chickens were fed on normal control (NC): 0 g flaxseed + no antioxidants sources, FS: 75 g flaxseed + no added antioxidant sources, VE8: 75 g flaxseed + 800 mg α-tocopherol/kg; DA8: 75 g flaxseed + 800 mg *D. angustifolia* extract/kg, TS8: 75 g flaxseed + 800 mg *T. schimperi* extract/kg; and CD8: 75 g flaxseed + 800 mg *C. domestica* extract/kg diets for 8 weeks. Hens were provided with 165 g feed/day and water ad libitum. They were vaccinated according to the recommended schedule and provided with 15 h lighting during laying. The chemical composition, nutrient levels, and fatty acid composition of the diets are provided in Supplementary Table S1. 

### 2.2. Chemicals and Materials

Chemicals: Vitamin E (α-tocopherol acetate, Shaanxi Bolin Bio-tec, China), Methanol (FineChem Limited, India), Ascorbic acid (Himedia, SD FineChem, India), Stearidonic acid (C18:4*n* − 3, Larodan, Limhamn, Sweden) and QuantiChrom TBARS Assay kit (DTBA-1000, BioAssay Systems, Hayward, CA, USA) and Conjugated linoleic acid (C18:2cis-9, trans-11, Matreya, State College, PA, USA). ABTS (2, 2′-azinobis-3-ethylbenzothiazoline-6-sulfonic acid), DPPH, (2, 2-diphenyl-1-picrylhydrazyl), Gallic acid, Trolox (6-hydroxy-2, 5, 7, 8-tetramethyl-chroman-2-carboxylic acid), Fatty acid methyl esters (FAMEs), Adrenic Acid (C22:4*n* − 6) were purchased from Sigma-Aldrich, Germany.

Materials: Lab mill (Model: FW100, China), Filter paper (Whatman No.1, England), Incubator Shaker (Shanghai ZHICHENG Analytical 103B), Rotary Evaporator (Stuart Bibby Scientific, RE300DB), Drying oven, (OVI/125/55/F/DIG/A, Genlab limited Widnes, Cheshire, UK), Freeze dryer (Mini Lyodel, DHPG-222, India), UV-Vis spectrophotometer (PerkinElmer, Lamda 950, UV/VIS, UK), Sarstedt tubes (Adelab Scientific, Thebarton, Australia), Digital Kitchen thermometer (DOQAUS, China), Centrifuge (ScanSpeed 40, LaboGene, Allerød, Denmark), Lab mill (Model A 11 Basic, IKA GmbH, Staufen, Germany), Bulk beads (Zirconium oxide Precellys beads, Bertin Instruments Technologies, Montigny-le-Bretonneux, France). Microwave Plasma-Atomic Emission Spectrometer (Agilent Technologies, 4200 MP-AES, USA), Homogenizer (Precellys Evolution, Bertin Instruments Technologies, Montigny-le-Bretonneux, France), Pyrex tubes (Pyrex, Hayes, UK), CP-Sil 88 CB column (100 m × 0.25 mm, Agilent, Santa Clara, CA, USA), PerkinElmer gas chromatograph CLARUS 680 (PerkinElmer Instruments, Waltham, MA, USA), 96-well-plate of the plate reader (SynergyTM MX, BioTek, Bad Friedrichshall, Germany), Food saver vacuum bag (Food Saver, PN:192465, Korea) and Water bath (Clifton, Nickel Electro Ltd., GB, Weston, UK).

### 2.3. Composition of Experimental Diets

The dry matter and the total nitrogen were determined according to [40], with Method 962.09. The calcium was analysed using Microwave Plasma-Atomic Emission Spectrometer as described by [41]. The crude fat was determined by Soxhlet extraction [42] (Method 920.39). The crude fiber was analysed by subsequent acidic and alkaline hydrolysis according to the procedure by [43]. The fatty acid composition of the diets was determined by gas chromatograph, with flame ionization detection (GC-FID), as described in Section 2.6.

### 2.4. Antioxidant Capacity of Plant Polyphenol Extracts (PPEs) and Diets

#### 2.4.1. Plant Extracts Preparation

The leaves, rhizomes of tested plants, and diet samples were powdered using a lab mill. Fifty milliliters of methanol was added to 5 g of powdered plant and feed samples in a conical extraction flask and shaken for 24 h at 25 °C. The solution was then filtered through a paper filter 3× to recover the extracts fully. Methanol was removed by a rotary evaporator followed by the concentration of the filtrate at 40 °C for 10 min using a drying oven. Methanol was completely removed using a freeze dryer, and the yield was calculated as per [44] and kept in the dark at 4 °C until use. 

#### 2.4.2. Phenolic Content of Plant Extracts and Diets

The total phenolic content (TPC) of the plant extracts and diets was determined as per [45], with slight modifications. From each diet, aliquots of 150 µL were taken in tubes from an initial 50 mg/mL and made up to 1000 µL (final concentration of 7.5 mg/mL). From each plant extract from an initial 50 mg/mL, 50 µL volume was taken and diluted to 1.5 mg/mL. Then, from 1.5 mg/mL aliquot of 400 µL for *T. schimperi* diluted to 1000 µL with a final concentration of 0.60 mg/mL. Similarly, 150 µL from 1.5 mg/mL for each *D. angustifolia* and *C. domestica* was taken in test tubes and diluted to 1000 µL, giving a final concentration of 0.225 mg/mL. Subsequently, 3.16 mL distilled water and 200 µL Folin-Ciocaltu reagent were added to each test tube containing plant and diet extracts. As a blank, methanol was added in place of the samples. After 8 min incubation at room temperature, 600 µL sodium carbonate (7.5%) solution was added and the solution was incubated at 40 °C for 30 min in the dark. Absorbance was recorded using a UV-Vis spectrophotometer at 765 nm against the blank. Gallic acid standard curves were developed for diet and plant extract samples. All determinations were conducted in triplicate. The equations obtained from the respective GA standard curves were used to calculate the total phenolic content as Gallic acid equivalent (GAE) per gram dry weight of the extracts and diets. 

#### 2.4.3. DPPH Radical Scavenging Capacities of Plant Extracts and Diets

The DPPH (2, 2-diphenyl-1-picrylhydrazyl) radical scavenging assay was carried out according to [46], with slight modification. In a flask covered with aluminum in the dark, 0.04 mg/mL DPPH was prepared. From each diet extract, with 50 mg/mL of initial concentration, 50 µL was taken and diluted to 1000 µL to provide a final concentration of 2.5 mg/mL. For the extracts of *T. schimperi*, *D. angustifolia*, and *C. domestica*, a concentration of 10, 50, 90, 130, and 170 to 210 μg/mL were prepared from an initial concentration of 1000 µg/mL. Then, 4 mL of 0.04 mg/mL of DPPH radical was added to tubes containing diet extracts at 2.5 mg/mL and plant extracts at different concentrations. One milliliter of methanol and 4 ml DPPH solution were prepared as blank. Reference curves were established from concentrations of ascorbic acid for diets and plant extracts. Test tubes were incubated for 1 hr at room temperature in the dark and absorbance was read at 517 nm using a UV-Vis spectrophotometer. All determinations were completed in triplicate. The capacities of plant and diet extract to scavenge DPPH radical as µM of ascorbic acid equivalent (AAE) were calculated from the ascorbic acid standard curve. In addition, the percentage of DPPH radical inhibition by various doses of the plant extracts were calculated by comparing the absorbance of the sample and the blank. The concentration plant extracts to inhibit 50% DPPH radical (IC_50_) values were generated by using the “drc package’ in R [47].

#### 2.4.4. ABTS Radical Scavenging Capacity of Plant Extracts and Diets

The ABTS (2, 2-azinobis-3-ethylbenzothiazoline-6-sulfonic acid) assay was applied to measure the antioxidant capacity of plant extracts and test diets as described by [48]. The ABTS radical cation was prepared by mixing equal volumes of 7 mM ABTS (0.0776 g/0.02 L) and 2.5 mM potassium persulfate (0.033 g/0.05 L) and incubated for 16 h in the dark. Then, ABTS radical stock solution was diluted with methanol 1:60 *v*/*v* until the absorbance value remained between 0.800 to 0.100 at 734 nm. Aliquots of 10 µL, from each diet extract with 50 mg/mL initial concentration, were diluted to 1000 µL to bring the final concentration to 0.5 mg/mL. For extracts of *T. schimperi, D. angustifolia,* and *C. domestica* a concentration of 5, 20, 35, 50, 65, and 80 μg/mL were prepared from an initial concentration of 1000 μg/mL. Subsequently, 3 mL of diluted ABTS radical was added to the test tubes containing 0.5 mg/mL diet and plant extracts at various concentrations and incubated for 5 min at 30 °C. Trolox was used as a standard antioxidant in similar concentrations to the plant extracts. As a blank, a mixture of 3 mL ABTS radical solution and 1 mL methanol was used. Absorbance was measured at 734 nm using a UV-Vis spectrophotometer. Reference curves were established from different concentrations of Trolox. The antioxidant capacities of the plant and diet extracts to scavenge ABTS radical as µM Trolox equivalent antioxidant capacity (TEAC) were calculated using the equations established from Trolox calibration curves. The plant extract’s capacity to scavenge ABTS radical as a percentage of inhibition was calculated after subtracting the absorbance of the sample from the blank. The specific dose for each plant extract to inhibit 50% ABTS radical (IC_50_) was determined using the “drc package” in R [47].

### 2.5. Breast Muscle Sampling and Processing 

At the end of the 8-week feeding trial, two out of three hens per replicate, (14 per treatment) were killed by cervical dislocation and bleeding [49]. Scalding followed at 65 °C for 1–2 min prior to plucking [50]. Once eviscerated manually, intact breasts were separated by incision at the fat line that differentiates it from the ribs and individually kept in food saver Ziplock bags for 12 h at 4 °C. Then, a 20 g sample was taken from different breast locations and kept in labelled Sarstedt tubes and stored at −18 °C until analysis. Cooked breast samples were prepared by putting 50 g of raw breast in small sized food-saver vacuum bags and cooked at 90 °C in a water bath until the internal core temperature reached 85 °C [51]. The internal temperature of the breast meat during cooking was monitored with a digital kitchen thermometer. 

### 2.6. Fatty Acid Analysis 

#### 2.6.1. Lipid Extraction and Transesterification of Diets and Breast Muscle

Chicken diets

The frozen diet samples were finely ground using liquid nitrogen in a mortar with a pestle. For extraction and direct fatty acid methylation of diet samples, a modified method from [52] was used. The samples were treated with 2 mL toluene (containing 19:0 methyl ester as internal standard) and 4 mL of 5% methanolic HCl. The mixture was shaken in a water bath at 60 °C for 2 h. After cooling, 10 mL of 6% K_2_CO_3_ solution was added and vortexed. All solvents used for feed lipid extraction contained 0.005% (*w*/*v*) of t-butylhydroxytoluene (BHT) to prevent oxidation of PUFAs. Next, the solutions were centrifuged at 4 °C, 1200× *g* for 5 min and finally the fatty acid methyl esters (FAMEs) were extracted two times each with 2 mL of *n*-hexane. After drying (1 g Na_2_SO_4_) and cleaning of the organic phase, if necessary with activated charcoal, the extracts were filtrated and evaporated using a vacuum centrifuge at 438 g at 30 °C for 30 min. Finally, the extracts were stored at −18 °C until GC analysis [53].

Chicken breast muscle (raw, cooked)

The frozen breast muscle samples (raw, cooked) were cut into small pieces and homogenized using a lab mill. For lipid extraction, approximately 1 g of breast muscle was weighed. Each Precellys-tube contained 20 pieces of 2.8 mm bulk beads and 2 pieces of 5 mm bulk beads. After the addition of 3 mL methanol and nonadeconoic acid (19:0) as an internal standard, the extracts (in duplicate) were homogenized 3 times at 25 s intervals at 4 °C and 6500 rpm using a homogenizer [54]. The homogenates were vortexed and transferred to Pyrex tubes containing 8 mL of chloroform. Then, the Precellys-tubes were washed two times with 1 mL methanol. All solvents used for breast muscle lipid extraction contained 0.005% (*w*/*v*) of t-butylhydroxytoluene (BHT) to prevent the oxidation of PUFAs.

After filtration, the lipid extracts of muscle samples were stored at 5 °C for 18 h in the dark and subsequently washed with a 0.02% CaCl_2_ solution. The organic phase was separated and dried with a mixture of Na_2_SO_4_ and K_2_CO_3_ (10:1, *w*/*w*), and the solvent was subsequently removed using a vacuum centrifuge at 438 g/min, 30 °C, 30 min. The lipid extracts were redissolved in 300 μL of toluene, and a 25 mg aliquot was used for methyl ester preparation [53]. Briefly, for transmethylation, 2 mL of 0.5 M sodium methoxide in methanol was added to the lipid extracts, which were shaken in a 60 °C water bath for 10 min. Subsequently, 1 mL of 14% boron trifluoride in methanol was added to the mixture, which was then shaken for an additional 10 min at 60 °C. The fatty acid methyl esters (FAMEs) were extracted twice with 2 mL of *n*-hexane and stored at −18 °C until high-resolution gas chromatography (HR-GC) analysis.

#### 2.6.2. Gas Chromatography Analysis

The fatty acid analysis of all sample extracts was performed using capillary high-resolution gas chromatography (HR-GC) with a CP-Sil 88 CB column. The GC column was installed in a PerkinElmer gas chromatograph CLARUS 680 with a flame ionization detector and split injection as described earlier [53]. Briefly, hydrogen was used as the carrier gas at a flow rate of 1 mL × min^−1^ while the split ratio was 1:20, with the injector and detector set at 260 °C and 280 °C, respectively. The GC oven temperature program was 150 °C for 5 min; heating rate of 2 °C/min until 200 °C and kept for 10 min; heating rate of 1 °C/min until 225 °C and kept for 20 min. For the calibration of the reference standard mixture “Sigma FAME,” the methyl ester of C18:1cis-11, C22:5*n* − 3, and C18:2cis-9, trans-11, C22:4*n* − 6, and C18:4*n* − 3 were used. The five-point calibration of single fatty acids ranged between 16 and 415 µg/mL and was assessed after GC analysis of five samples. Fatty acids were displayed as a concentration in mg/100 g of tissue.

### 2.7. Analysis of Oxidative Stability in Raw and Cooked Breast Meat

The frozen breast muscle samples (raw, cooked) were cut into small pieces and homogenized using a lab mill. For the analysis of oxidative stability, approximately 400 mg of muscle sample was weighed in a tube. Each 7 mL Precellys-tube contained 5 pieces of 2.8 mm bulk beads and 1 piece of 5 mm bulk beads. The sample preparation and measurement of oxidative stability in breast muscle samples was performed according to the guidelines of the assay QuantiChrom TBARS Assay kit. After the addition of 2 mL of ice-cold phosphate-buffered saline (PBS) solution (200 mL and 133 µL BHT), the extracts (in triplicate) were homogenized 2 times at 10 s intervals at 4 °C and 6500 rpm using a homogenizer. 

Then 200 µL of ice-cold 10% trichloroacetic acid (TCA) solution was added and incubated for 5 min on ice. After that, the sample extracts were centrifuged for 5 min at 14,000 *g*, and 200 µL of thiobarbituric acid (TBA) was added to 200 µL sample extract vortexed and incubated for 60 min at 100 °C. After cooling to room temperature, 100 µL of the sample extracts were transferred into the 96-well-plate of the plate reader. Then, the colour intensity (OD) was measured at 535 nm. The standard solutions of malondialdehyde (MDA) were prepared at a concentration range from 0.0 to 1.5 µM MDA and the colour intensity was measured using the same procedure as for the sample solutions. The concentrations of thiobarbituric acid reactive substances (TBARS) were calculated using the MDA standard calibration. Finally, the TBARS concentration was expressed in µg MDA/g of breast muscle.

### 2.8. Calculating Lipid Health Indices 

The potential health contribution of lipids can be calculated by considering the specific fatty acids and their groups [36]. In this study, the lipid health indices such as *n* − 6/*n* − 3 PUFA ratio, saturation indices as saturated and unsaturated fatty acids ratio (s/p), atherogenic index (AI), thrombogenic index (TI) and hypocholesterolemic/hypercholesterolemic (h/H) ratio were estimated. The AI, *n* − 6/*n* − 3 PUFA, s/p ratios, the total sum of fatty hypercholesterolemic fatty acids (HFA), and desirable fatty acids (DFA) were calculated as per the below Equations (1)–(4) [23]. Moreover, TI, h/H ratio, and the nutritional value indices (NVI) were determined according to Equations (5)–(7) as suggested by [36].

(1) $$AI=\frac{\left(4\times C14:0+C12:0+C16:0\right)}{\left[\sum MUFA+\sum^{\;}n6\;PUFA+\sum^{\;}n3\;PUFA]\right]}$$

(2) $$s/p=\frac{\left(C14:0+C16:0+C18:0\right)}{\left(MUFA+PUFA\right)}$$

(3) HFA=C12:0+ C14:0+C16:0

(4) AFA=C18:0+ UFA

(5) $$h/H=\frac{\left(C18:1n9+C18:2n6+C18:3n3+C20:4n6+C20:5n3+C22:5n3+C22:6n3\right)}{\left(C14:0+C16:0\right)}$$

(6) $$TI=\frac{\left(C14:0+C16:0+C18:0\right)}{[\left(0.5\times MUFA\right)+\left(0.5\times n-6\;PUFA+\left(3\times n-3\;PUFA\right)+\left(\frac{n-3\;PUFA}{n-6\;PUFA}\right)\right]}$$

(7) $$NVI=\frac{C18:0+C18:1}{\;C16:0}$$

where: *PUFA*—polyunsaturated fatty acids; *MUFA*—monounsaturated fatty acids; *UFA*—unsaturated fatty acids.

### 2.9. Statistical Analysis

Data were analysed using the R platform [55] and RStudio environment for statistical computing [56], and the “lme4” package [57] to perform a linear mixed model. The linear mixed model was used to predict the fatty acid concentration as a response variable (RS) including room and diets as fixed effects and pen as random effects. The distribution of residuals was visually inspected using the plot function in R to assess the assumptions of the analysis (i.e., linearity, independence, homoscedasticity, normality of residuals). The “emmeans” package [58] was applied to produce the estimated marginal means for each diet with 95% confidence intervals. Multiple paired comparisons between treatments were made on overall significant effects by using Tukey adjusted *p*-values. In addition, using the “drc” package in R [47], the concentration required to inhibit 50% radicals (IC_50_) values were estimated for each extract by applying the radical scavenging percentages of the plant extracts as a response variable.

## 3. Results and Discussion

### 3.1. Phenolic Content and Antioxidant Activities of Plant Extracts

The phenolic content and antioxidant capacity of the methanolic plant extracts are reported in Table 1. Accordingly, the extracts of *C. domestica* and *D. angustifolia* had higher (*p* < 0.05) total phenolic content (TPC) compared to *T. schimperi*. The TPC obtained in the extract of *C. domestica* agreed with the ones reported from India for *Curcuma longa* [59] but was higher compared to the ethanolic extract reported by [60]. The observed TPC in *the* methanolic extract of *T. schimperi* in the present study agreed with the report of [61] but was higher compared to the reported using methanolic-aqueous extraction solvents [62]. However, the TPC in *D. angustifolia* was lower compared to the one previously reported [27].

Among the three plant extracts, *T. schimperi* and *D. angustifolia* had higher (*p* < 0.05) efficiency in scavenging DPPH radical with respective IC_50_ values of 33.97 and 25.59 µg/mL than the *C. domestica* extract (*p* < 0.05) (Table 1). Here, higher antioxidant capacity and lower IC_50_ were observed in the methanolic extract of *T. schimperi* compared to the report by [63]. The *D. angustifolia* methanolic extract had a comparable IC_50_ value of 25.59 µg/mL with ascorbic acid to scavenge DPPH free radical (*p* > 0.05). The IC_50_ values of *D. angustifolia* agreed with the previously reported ones [27]. Similarly, the IC_50_ values of *T. schimperi* and *D. angustifolia* were comparable with ascorbic acid and Trolox reference antioxidants in scavenging DPPH and ABTS radicals, respectively (*p* > 0.05). 

However, *C. domestica* had the lowest antioxidant capacity, while having a relatively higher phenolic content (Table 1). This could be because the deep yellow colour in *C. domestica* extract might be associated with higher phenolic content and decreased antioxidant power, as other studies found a positive correlation between colour with total phenolic content and a negative correlation with antioxidant capacity [64,65]. Moreover, the extract of *C. domestica* had lower (*p* < 0.05) Ascorbic Acid Equivalent (AAE) antioxidant capacity towards scavenging DPPH radicals compared to those in *T. schimperi* and *D. angustifolia* extracts with 221.81 and 236.53 µM AAE/mg of dry extracts, respectively (Table 1). Overall, as per the observed current phenolic content (72–277 mg GAE/g) with lower IC_50_ values to scavenge DPPH and ABTS radicals, the plant extracts can be considered a good source of natural antioxidants. Related to this, improved antioxidant capacities and decreased MDA levels were reported in broiler chickens fed *Alpinia galangal* rhizomes extract at 500 or 750 mg/kg diet [66].

### 3.2. Phenolic Content and Antioxidant Activities of Test Diets

The phenolic concentration and antioxidant capacity of the test diets are reported in Table 2. The test diets total phenolic contents and antioxidant capacities obtained were higher (*p* < 0.05) in diets supplemented with *D. angustifolia, T. schimperi*, and *C. domestica* extracts (DA8, TS8, CD8), compared to those with no antioxidant sources (FS, NC). Furthermore, the equivalent antioxidant power obtained between plant extracts and vitamin E supplemented diets (*p* > 0.05) in scavenging DPPH free radicals. However, the vitamin-E supplemented diet was superior (*p* < 0.05) in scavenging ABTS radicals compared to plant extract-containing diets (DA8, TS8, and CD8) (Table 2). 

Chickens face oxidative stress from various factors such as the inclusion of higher dietary fats [67]. The supplementation of suitable natural antioxidants in animal feed is recognized as an important nutritional intervention to avoid or reduce oxidative stress [20,68] and to enhance the nutritional value of meat and for consumers’ health benefits [69]. Overall, the observed increase in phenolic content and radical scavenging activities in diets supplemented with plant extracts suggest their potential use to combat oxidative stress in chickens. Moreover, phenolic compounds are reported to enhance the activities of dismutase and glutathione peroxidase enzymes and to reduce lipid peroxidation in meat [70].

### 3.3. Fatty Acid Contents of Raw Breast Muscle 

The effects of feeding flaxseed, along with plant extracts and/or vitamin E on selected fatty acid contents in raw breast muscle of Sasso chickens, are presented in Table 3, and values for all investigated fatty acids are provided in Supplementary Table S2. The dietary treatments had no significant (*p* > 0.05) effect on breast muscle fat content. Previously, a fat-lowering effect in breast muscle was reported in broilers fed with flaxseed and turmeric rhizome powder [23]. Among the saturated fatty acids (SFAs), C14:0 and C16:0 were reported to be the most atherogenic agent [71], in this study feeding plant extracts had no effect (*p* > 0.05) on breast muscle C14:0 and C16:0 contents. Among the extracts, TS8-fed hens had the lowest C4:0 and C18:0 content in breast muscle. The SFAs composition was reported to be less affected by dietary modification of the chicken diet compared to PUFA [72,73]. In this study, the C14:0 and C16:0 content in the breast muscle were lower compared to the amount reported, in broiler chickens fed with 10% flaxseed and turmeric powder [23].

Moreover, no significant (*p* > 0.05) difference was observed in monosaturated fatty acids (MUFAs) contents between the tested diets. Previous reports were inconsistent regarding breast muscle MUFA content, in that [74] found no effect, while [75] reported a decreasing trend in chickens fed flaxseed. In another study, a significant increase in breast MUFA was observed when chickens were fed flaxseed meals and grapeseed meal supplements [76]. However, no difference was reported in breast MUFA in broiler chickens fed with flaxseed and various amounts of turmeric rhizome powder [23]. In this study, the breast muscle total *n−6* PUFAs, C18:2*n* − 6, and C20:4*n* − 6 contents were not affected (*p* > 0.05) in any of the diets. Moreover, the values are comparable to the previous report by [23] in broiler chickens fed with 100 g flaxseed and turmeric rhizome powder. In contrast, no difference in breast muscle *n* − 6 PUFA and LA and an increase in C20:4*n* − 6 were reported in broiler chickens fed with 2 g linseed oil and 2 g sweet chestnut tannins [77].

Feeding hens with flaxseed along with extracts of *C. domestica* and *T. schimperi* significantly (*p* < 0.05) increased the level of ALA in breast muscle compared to the FS diet. Moreover, there was no significant difference observed (*p* > 0.05) in breast muscle ALA concentrations between hens fed with plant extract and vitamin E supplemented diets. Previous studies reported an increase [78] or no difference [34] in breast muscle ALA in broiler chickens fed with 0.1% pomegranate extract and *Amphora coffeaeformis* in linseed oil-enriched diets, respectively. In contrast to the present study, feeding vitamin E along with 20% linseed oil increased the breast muscle ALA compared to diets supplemented with sweet chestnut tannins [77].

The breast muscle C20:5*n* − 3 (EPA) content was highest in CD8 (1.49 mg/100 g) and lowest in the NC diet (0.31 mg/100 g) (Table 3). Feeding hens with FS doubled the breast EPA and increased significantly (*p* < 0.05) the C22:6*n* − 3 (DHA) content in breast muscle in comparison to the NC diet (Table 3). This likely follows the higher ALA content in FS compared to the NC diet as a precursor for its conversion to EPA and DHA. Compared to hens fed with FS, adding plant extracts increased the breast muscle EPA content by 74% in TS8, 77% in DA8, and 125% CD8 diets. Similar increase in breast muscle EPA content by 106% was observed in VE8 compared to the FS diet. Between hens fed with NC and FS diets, no difference (*p* > 0.05) was observed in breast muscle DPA (C22:5*n* − 3) content. However, the breast muscle DPA (C22:5*n* − 3) content was significantly increased (*p* < 0.05) in hens fed with TS8 and CD8, VE8 compared to NC and FS diets. Interestingly, the DPA concentration was the same between hens fed with plant extract incorporated feeds and vitamin E containing diet (*p* > 0.05) (Table 3). Similarly, the FS diet fed hens had a higher content of C22:6*n* − 3 (DHA) in breast muscle compared to the NC diet (*p* < 0.05). Moreover, feeding chickens with CD8, but not the DA8 and TS8 diets, significantly (*p* < 0.05) increased the breast muscle DHA content compared to the FS diet. However, feeding chickens with DA8, TS8, and CD8 diets resulted in a significantly (*p* < 0.05) higher DHA content in the breast muscle compared to the NC diet (Table 3). The observed increase in breast muscle EPA (DA8, TS8, and CD8) and DHA (CD8) diets might indicate their potential to stimulate the conversion efficiency of dietary ALA precursor into its longer counterparts.

Increasing the EPA and DHA contents is one of the most desired attributes of meat functionalization [79], these nutrients are components of heart-healthy diets [80]. Hence, the effect of the inclusion of flaxseed in the chicken diet to increase the breast muscle EPA and DHA content has been tested [80,81,82,83]. Previously, feeding 500 mg sage extract/kg diet as an antioxidant was found to increase DHA and other *n* − 3 PUFAs in breast meat [84]. Other studies observed an enhanced conversion of C18:3*n* − 3 (ALA) to LC *n* − 3 PUFA in chicken fed with plant extracts [85] and plant leaves in meal/powder forms [21,36,75] in a flaxseed diet. The observed increase in breast muscle EPA and DHA contents in feeding plant extracts in the present study were comparable with the reported increase in breast EPA and DHA following the inclusion of turmeric rhizome powder [23] and sweet chestnut tannins [77] as an antioxidant in flaxseed enriched broiler chicken diets.

Overall, feeding hens with FS significantly (*p* < 0.05) increased the *n* − 3 PUFA in breast muscle compared to the NC diet. The CD8 fed hens, but not the DA8 and TS8 diets had a significantly (*p* < 0.05) higher *n* − 3 PUFA content in breast muscle compared to the FS diet. Consistent with our findings, Kumar [23] reported higher *n* − 3 PUFA in the breast muscle of broilers fed with flaxseed along with turmeric rhizome powders. In contrast, no difference [86] or a decrease [87] in breast muscle *n* − 3 PUFA was reported in broilers fed with 2% grapeseed meal and 1.5–5% grapeseed oil in flaxseed enriched diets, respectively. The differences in breast *n* − 3 PUFA observed in this and across previous studies may be associated with plant species differences in active components. In the breast muscle, no difference (*p* > 0.05) in PUFA content was observed between treatment groups. Similarly, studies have shown no variation in breast PUFA in broiler chickens fed with flaxseed/linseed oil along with 0.5% of curry leaf, ginger or turmeric powders [88], 5–12.5% turmeric powder [23], and 2 g sweet chestnut tannins [77].

The comparison of the current results showed no difference (*p* > 0.05) in breast muscle EPA, DHA, and *n* − 3 PUFA contents between hens fed with vitamin E and plant extracts supplemented diets. However, feeding the hens with vitamin E elevated (*p* < 0.05) the breast muscle concentrations of EPA and DHA compared to those fed the FS diet. This might indicate the role of vitamin E and plant extracts as an antioxidant for preventing lipid peroxidation in feed and reducing oxidative stress in chickens. In agreement with our results, no difference in breast muscle EPA and DHA content was found between broiler chickens fed with 200 mg of vitamin E and 2 g sweet chestnut as natural antioxidants [77]. However, the same study also reported a positive effect of supplementing vitamin E in enhancing breast muscle EPA and DHA content compared to a diet with only flaxseed [77].

### 3.4. Fatty acid Content in Cooked Breast Muscle 

The fatty acid concentration of the cooked breast muscle is presented in Table 4 and full lists of analysed fatty acid concentrations are provided in Supplementary Table S3. Cooking improves nutrient digestibility, enriches the taste, and softens the texture and safety of meat. However, non-optimized cooking might lead to undesirable effects on nutritional properties and the formation of heat-induced toxicants [89]. The loss of beneficial *n* − 3 LC PUFAs in enriched meat has been reported [6,90], and thus, the quantification of fatty acids in cooked meat is required [91]. To increase the stability, contain the losses of *n* − 3 PUFA, and reduce lipid peroxidation during meat processing, the use of natural antioxidants has been suggested [92]. Until now, however, there were few studies which were evaluating the dietary plant extracts in a flaxseed-enriched chicken diets for their possible effect on the content of LC *n* − 3 PUFAs in cooked meat.

In this study, a higher (*p* < 0.05) content of heat-susceptible *n* − 3 LC-PUFAs, such as EPA (C20:5*n* − 3) and DHA (C22:6*n* − 3), were retained in cooked breast muscle in hens fed with FS compared to the NC diet (Table 3). It supports that the low EPA and DHA in breast muscle from hens fed NC further diminished as a consequence of cooking. Feeding hens with plant extract (TS8 and CD8) retained higher (*p* < 0.05) EPA and DHA contents in cooked breast muscle compared to the FS diet. Henceforth, plant extract inclusion as an antioxidant might prevent the loss of oxidative susceptible and heat-sensitive EPA and DHA in breast muscle. Moreover, the EPA, DHA, and total *n* − 3 PUFA content in the cooked breast muscle from hens fed plant extracts were comparable to the ones fed with vitamin E (*p* > 0.05).

### 3.5. Lipid Health Indices and Oxidative Stability in Raw and Cooked Breast Muscle

Lipid health indices

The lipid health indices and oxidative stability results in raw and cooked breasts are presented in Table 5. Lowering the *n* − 6/*n* − 3 PUFA ratio is considered as a crucial dietary intervention to prevent several health disorders [80], but achieving the recommended ratio of 5:1 in human diets remains one of the main challenges in modern food production systems [93,94]. In this study, a significantly lower (*p* < 0.05) *n* − 6/*n* − 3 ratio was observed in both raw and cooked breast muscle from chickens fed the FS diet compared to the NC diet (Table 5). This implies a higher *n* − 3 PUFA in FS than in the NC diet.

In the cooked breast muscle, SFAs, C14:0, C16:0, and C18:0 contents were not significantly (*p* > 0.05) different between treatment groups. The breast muscle total MUFA, C16:1cis-9 and C18:1cis-9 contents were the highest in hens fed with DA8, but differences were not significant (*p* > 0.05) between the dietary groups. Similarly, the concentrations of LA (C18:2*n* − 6), ALA (C18:3*n* − 3), and DPA (C22:5*n* − 3) in cooked breast muscle did not differ significantly (*p* > 0.05) between the treatment groups. A recent comparative study in slow-growing leghorn chickens, reported a significant loss in breast muscle *n* − 3 PUFA during cooking, whereas the saturated fatty acids (C14:0, C16:0, and C18:0), as well as unsaturated fatty acids such as LA, ALA, and DPA contents, were less affected [6]. Furthermore, [90] reported no difference (*p* > 0.05) in cooked meat fatty acid profile between chickens fed with flaxseed as well as different antioxidants. In contrast, a respective decrease of 6.2% and 6.8% in SFA and MUFA in cooked chicken meat was reported in [91].

Feeding hens with TS8 in both raw and cooked and DA8 in raw breast significantly (*p* < 0.05) lowered the breast *n* − 6/*n* − 3 ratio compared to the FS diet (Table 5). A decreasing effect on breast muscle *n* − 6/*n* − 3 ratio was reported in broilers fed with flaxseed along with turmeric rhizome powder [23] and upon supplementing phenolic antioxidant of pomegranate peel and pomegranate peel extract [72]. In addition, no significant difference in *n* − 6/*n* − 3 between hens fed with plant extract containing diets and VE8 (*p* > 0.05). In a similar study, no difference was observed in breast muscle *n* − 6/*n* − 3 PUFA ratio between broilers fed with linseed oil and vitamin E and those fed with sweet chestnut tannins [77]. In general, the *n* − 6/*n* − 3 ratio in the current finding was not lower than 4, which is the recommended ratio to prevent the occurrence of cardiovascular disorders [95]. Excess intake of diets with a high *n* − 6/*n* − 3 ratio may promote inflammatory, cancer, and cardiovascular risks [96].

As per our findings, feeding hens with FS significantly (*p* < 0.05) decreased the breast muscle thrombogenic indices (TI) compared to those fed with the NC diet (Table 5). However, there was no significant difference in atherogenic indices (AI) (*p* > 0.05) between treatment groups with both raw and cooked breast meats. Similarly, the thrombogenic indices (TI) of breast meat (both raw and cooked) of chickens fed with plant extract containing diets and FS were not significantly different (*p* > 0.05). The AI, TI, and h/H ratio are health and nutritional value indicators of lipids [97]. The AI is a major indicator of cardiovascular risk [98], and its lower value suggests that consumption of such meat could potentially contribute to a decrease in cardiovascular risk. The current AI and TI values in both raw and cooked breast muscle were less than 1, which is in the recommended range to provide health benefits [99]. According to [97], diets with a higher TI promote the formation of clots in blood vessels. A decrease in TI values in foods is associated with lower health risks [99,100]. Similar TI values to the present study were reported in broilers fed on similar feeding protocols [23].

In this study, the h/H ratio, saturation indices (s/p), nutritional value indices (NVI), desirable fatty acids (DFA), and hypercholesterolemic fatty acids (HFA) in both raw and cooked breast muscle did not vary between dietary treatments. Comparable NVI, DFA, and HFA values were reported by [23] in similar dietary protocols. Feeding 4% flaxseed along with various levels of grape seed oil as an antioxidant source did not affect the breast muscle h/H ratio [87]. In contrast, feeding broiler chickens with turmeric rhizome powder and flaxseed was found to increase the h/H ratio and DFA and a decrease in HFA and s/p in breast muscle [23]. A previous study also found no effect of cooking in meat s/p ratio from broiler chickens fed diets supplemented with different fat types [91].

Oxidative stability

Oxidative deterioration in meat products is associated with the in vivo exposure of chicken to various stressors including diet. At the molecular level, excess oxidative stress lead to oxidative damage to important biological molecules including lipids [101]. In this study, as a major secondary lipid peroxidation product, the malondialdehyde (MDA) value varied from 1.53 to 2.60 µg/g and 9.28 to 16.92 µg/g in raw and cooked breast muscles, respectively (Table 5). However, the MDA concentration in raw breast muscle was significantly lower (*p* < 0.05) in hens fed with plant extract antioxidant sources (DA8, TS8, CD8) compared to those fed with FS and the NC diets. A decrease in MDA concentrations by 52.04, 53.84, and 69.93% was observed in hens fed TS8, DA8 and CD diets, respectively, compared to the FS diet. Hens fed plant extracts had lower MDA values (1.53 to 1.71 µg/g) in raw breast muscle compared to fed control diets (2.00 to 2.60 µg/g). These lower MDA values could be attributed to the protective effects of plant polyphenol extracts against lipid peroxidation, as meat products with less than 3 mg MDA/kg sample are considered to be preserved from oxidative changes [10]. In cooked breast, a 5–8 fold increase in MDA was observed compared to raw breast muscle. A comparable 3–5 fold MDA increase in *n* − 3 PUFA enriched cooked breast was reported by [10]. Overall, plant extracts decreased lipid peroxidation in both raw and cooked breast but were found to be more efficient in raw than in cooked breast muscle. Here, an increase in cooked breast MDA values was observed but lower in those fed plant extracts. A significant decrease (*p* < 0.05) in both raw and cooked breast MDA was obtained in hens fed TS8 compared to the FS diet. Hence, an increase in MDA values in cooked breast muscle from hens fed diets with no antioxidant sources (FS, NC), indicates a lack of sufficient antioxidants to prevent lipid peroxidation. A rapid increase in lipid peroxidation has been observed in chicken meat heated at 70 °C and stored for 2 to 4 days [102].

Generally, the inclusion of plant extracts was found to reduce lipid peroxidation as observed in a previous study [103]. Moreover, the combined inclusion of linseed oil with levels of pine needles powder [104] and 3% grape seed oil [87] in broiler diets was found to decrease the MDA content in breast muscle. Furthermore, the inclusion of 4% linseed oil and various natural antioxidants in broiler diets such as rosemary, green tea, grape seed, and tomato extracts resulted in a striking difference in preventing lipid peroxidation in broiler meat but with less effect than following the addition of vitamin E [105]. Interestingly, in this study, there was no difference between hens fed with plant extracts and vitamin E in breast muscle MDA concentration (*p* > 0.05). In agreement with the present finding, no difference was found in breast muscle MDA value between broilers fed with 2 g sweet chestnut tannins and 200 mg vitamin E as antioxidants in a linseed oil-enriched diet [77].

## 4. Conclusions

Feeding hens with plant extract in CD8 and VE8 significantly increased DHA and EPA contents, while DA8 and TS8 elevated EPA in raw breast muscle. While the SFA and MUFA contents were not influenced, a higher content of EPA and DHA was retained in cooked breast muscle (DA8, TS8, and CD8 diets). Moreover, feeding hens with diets without plant polyphenol extracts (FS, NC) increased the lipid peroxidation in raw breast muscle, whereas the inclusion of plant extracts decreased it. Feeding plant extracts in DA8, TS8, and CD8 diets showed superior efficiency in decreasing lipid peroxidation in breast muscle compared to feeding with vitamin E supplements. Further research is needed to verify whether the observed increase in *n* − 3 PUFA in breast muscle is a direct consequence of plant extracts inclusion in the diet or it rather indirectly resulted from the reduction of oxidative stress. 

## Figures and Tables

**Table 1:** Total phenolic content, DPPH, and ABTS radical scavenging capacities of plant extracts.

Extracts/ References	Total Phenolic Content (mg GAE/g)	IC_50 (DPPH)_ (µg/mL)	IC_50 (ABTS)_ (µg/mL)	DPPH (µM AAE/mg)	ABTS (µM TEAC/mg)
*T. schimperi*	72.34 ± 15.85 ^b^	33.97 ± 4.35 ^a^	6.75 ± 0.39	221.81± 6.96 ^a^	5332.60 ± 230.88
*D. angustifolia*	260.82 ± 45.29 ^a^	25.59 ± 4.10 ^ba^	8.81 ± 0.76	236.53 ± 10.62 ^a^	5164.73 ± 277.12
*C. domestica*	277.64 ± 16.66 ^a^	96.98 ± 5.37 ^c^	13.45 ± 6.54	116.01 ± 20.84 ^b^	5017.05 ± 102.80
*Ascorbic acid*	-	16.40 ± 0.04 ^b^	-	-	-
*Trolox*	-	-	8.00 ± 4.01	-	-

Data are expressed as mean + Standard Deviation (SD) (*n* = 3). Mean values within a column with different ^abc^ superscript letters represent significant differences (*p* < 0.05). IC: Inhibition concentration; DPPH: 2, 2-diphenyl-1-picrylhydrazyl; ABTS: 2, 2′-azinobis-3-ethylbenzothiazoline-6-sulfonic acid, AAE: Ascorbic acid equivalent antioxidant capacity; TEAC: Trolox equivalent antioxidant capacity.

**Table 2:** Phenolic contents, DPPH, and ABTS radicals scavenging capacities of test diets.

Diets	Total Phenolic Content (mg GAE/g)	DPPH (µM AAE/mg)	ABTS (µM TEAC/mg)
Normal control + no antioxidants (NC)	6.86 ± 0.79 ^a^	6.91 ± 0.38 ^a^	1.80 ± 0.17 ^a^
75 g flaxseed + no antioxidants (FS)	5.60 ± 0.81 ^a^	4.96 ± 0.05 ^b^	1.46 ± 0.52 ^ad^
75 g flaxseed + 800 mg vit E (VE8)	12.38 ± 0.42 ^b^	9.63 ± 0.54 ^c^	5.69 ± 0.21 ^b^
75 g flaxseed + 800 mg DA (DA8)	12.97 ± 1.06 ^b^	9.19 ± 0.56 ^c^	3.70 ± 2.43 ^c^
75 g flaxseed + 800 mg TS (TS8)	14.68 ± 1.53 ^b^	9.30 ± 0.50 ^c^	2.75 ± 1.04 ^cd^
75 g flaxseed + 800 mg CD (CD8)	10.77 ± 0.67 ^b^	8.94 ± 1.04 ^c^	2.89 ± 0.21 ^cd^

Data are expressed as mean + Standard Deviation (SD) (*n* = 3). Mean values within a column with ^abcd^ different superscript letters represent significant differences at *p* < 0.05. GAE: Gallic acid equivalent; DPPH: 2, 2-diphenyl-1-picrylhydrazyl; AAE: Ascorbic acid equivalent; ABTS: 2,2-azinobis (3-ethilenzotianzolin)-6-sulfonate; TEAC: Trolox equivalent antioxidant capacity; Diets: NC: Normal control (0 g flaxseed + no antioxidant sources), FS: Flaxseed (75 g flaxseed + no added antioxidants; VE8: Positive control (75 g flaxseed + 800 mg vitamin-E/kg); DA8: 75 g flaxseed + 800 mg *Dodonaea angustifolia*/kg; CD8: 75 g flaxseed + 800 mg *curcuma domestica*/kg; and TS8: 75 g flaxseed + 800 mg *Thymus schimperi*/kg diet.

**Table 3:** Effect of supplementing plant polyphenol extracts and flaxseed on fat content and fatty acid concentration (mg/100 g) in raw breast muscle of Sasso chickens.

Fatty Acids (mg/100 g)	^1^ Treatments												Random Effect	*p*-Value
	NC		FS		VE8		DA8		TS8		CD8			
	Mean	95% [LCL, UCL]	Mean	95% [LCL, UCL]	Mean	95% [LCL, UCL]	Mean	95% [LCL, UCL]	Mean	95% [LCL, UCL]	Mean	95% [LCL, UCL]		
C14:0	7.41	[5.87, 8.94]	7.43	[5.90, 8.97]	8.37	[6.84, 9.90]	8.17	[6.64, 9.71]	6.37	[4.84, 7.90]	8.29	[6.76, 9.82]	0	0.3971
C16:0	297.0	[253, 342]	288.0	[244, 333]	319.0	[275, 364]	315.0	[270, 359]	262.0	[217, 306]	322.0	[277, 366]	0	0.3610
C18:0	114.0	[102.2, 126]	112.0	[99.7, 124]	119.0	[107.3, 131]	120.0	[108.3, 132]	101.0	[88.7, 113]	120.0	[107.9, 132]	0	0.1634
C16:1cis-9	20.8	[15.2, 26.5]	19.5	[13.9, 25.2]	26.1	[20.4, 31.7]	22.8	[17.2, 28.5]	19.0	[13.3, 24.6]	25.6	[20.0, 31.3]	0	0.3114
C18:1cis-9	326.0	[262, 390]	322.0	[258, 386]	358.0	[294, 422]	357.0	[293, 421]	287.0	[223, 351]	363.0	[299, 427]	0	0.4900
C18:2*n* − 6	279.0	[232, 327]	271.0	[224, 319]	266.0	[218, 313]	291.0	[244, 339]	231.0	[184, 279]	282.0	[234, 329]	0	0.5525
C18:3*n* − 3	16.90 ^a^	[11.1, 22.6]	20.80 ^ab^	[15.1, 26.6]	26.60 ^bc^	[20.9, 32.4]	27.90 ^bc^	[22.2, 33.7]	22.70 ^abc^	[16.9, 28.4]	30.50 ^c^	[24.8, 36.3]	0	0.0189
C20:4*n* − 6	84.20	[78.1, 90.4]	83.20	[77.0, 89.4]	86.50	[80.3, 92.7]	82.50	[76.3, 88.7]	79.90	[73.7, 86.0]	86.60	[80.4, 92.7]	non 0	0.6225
C20:5*n* − 3	0.31 ^a^	[0.003, 0.62]	0.66 ^a^	[0.35, 0.97]	1.36 ^b^	[1.05, 1.67]	1.17 ^b^	[0.86, 1.47]	1.15 ^b^	[0.84, 1.46]	1.49 ^b^	[1.17, 1.79]	non 0	<0.0001
C22:5*n* − 3	8.26 ^a^	[7.36, 9.15]	9.26 ^ac^	[8.37, 10.15]	11.40 ^b^	[10.51, 12.29]	10.42 ^bc^	[9.52, 11.31]	10.59 ^b^	[9.70, 1.49]	10.99 ^b^	[10.1, 11.88]	0	<0.0001
C22:6*n* − 3	23.80 ^a^	[21.1, 26.6]	34.70 ^b^	[31.9, 37.5]	39.60 ^c^	[36.8, 42.3]	34.60 ^bd^	[31.8, 37.4]	36.90 ^bcd^	[34.1, 39.7]	43.30 ^c^	[40.5, 46.0]	non 0	<0.0001
^2^ ∑SFA	430.0	[372, 489]	420.0	[361, 478]	458.0	[399, 517]	455.0	[397, 514]	381.0	[323, 440]	462.0	[403, 521]	0	0.3362
^3^ ∑MUFA	386.0	[311, 460]	378.0	[304, 452]	424.0	[349, 498]	418.0	[344, 493]	342.0	[268, 416]	430.0	[356, 504]	0	0.5033
^4^ ∑ PUFA	449.0	[395, 504]	448.0	[394, 503]	458.0	[403, 512]	475.0	[421, 530]	408.0	[354, 463]	482.0	[427, 536]	0	0.4594
∑ *n* − 3 PUFA	49.90 ^a^	[43.3, 56.6]	66.20 ^b^	[59.6, 72.9]	79.90 ^bc^	[73.3, 86.6]	75.20 ^bc^	[68.5, 81.8]	72.20 ^bc^	[65.5, 78.8]	87.3 ^c^	[80.7, 94.0]	non 0	<0.0001
∑ *n* − 6 PUFA	398.0	[348, 449]	381.0	[331, 432]	376.0	[326, 427]	399.0	[348, 450]	335	[285, 386]	393	[343, 444]	0	0.4642
Fat (%)	1.27	[1.09, 1.46]	1.25	[1.06, 1.44]	1.34	[1.15, 1.52]	1.35.0	[1.16, 1.53]	1.12	[0.93, 1.31]	1.37	[1.18, 1.55]	0	0.4040

Estimated marginal means (emmeans) with 95% upper confidence (UCL) and lower confidence limits (LCL); emmeans within a row with ^abcd^ different superscript letters are significantly different at *p* < 0.05. ^1^ NC: 0 g flaxseed + no antioxidant sources, FS: 75 g flaxseed + no antioxidant sources, VE8: 75 g flaxseed + 800 mg α-tocopherol/kg, DA8: 75 g flaxseed + 800 mg *D. angustifolia* extract/kg, TS8: 75 g flaxseed + 800 mg *T. schimperi*/kg, CD8: 75 g flaxseed + 800 mg *C. domestica*/kg diet; ^2^ Sum of saturated fatty acids; ^3^ Sum of monounsaturated fatty acids; ^4^ Sum of polyunsaturated fatty acids.

**Table 4:** Effect of supplementing plant polyphenol extracts and flaxseed on fat content and fatty acid concentration (mg/100 g) in cooked breast muscle of Sasso T451A chickens.

Fatty Acids (mg/100 g)	^1^ Treatments												Random Effect	*p*-Value
	NC		FS		VE8		DA8		TS8		CD8			
	Mean	95% [LCL, UCL]	Mean	95% [LCL, UCL]	Mean	95% [LCL, UCL]	Mean	95% [LCL, UCL]	Mean	95% [LCL, UCL]	Mean	95% [LCL, UCL]		
C14:0	12.15	[7.68, 16.6]	11.16	[6.69, 15.6]	12.71	[8.24, 17.2]	15.99	[11.52, 20.5]	7.98	[3.51, 12.4]	11.53	[7.06, 16.0]	non 0	0.2562
C16:0	456.0	[298, 613]	430.0	[273, 587]	478.0	[321, 635]	599.0	[441, 756]	322.0	[165, 479]	453.0	[296, 610]	non 0	0.2803
C18:0	152.0	[115.3, 189]	145.0	[108.4, 182]	163.0	[125.9, 200]	187.0	[149.8, 224]	125.0	[87.6, 162]	151.0	[114.3, 188]	non 0	0.3012
C16:1cis-9	46.5	[17.93, 75.1]	43.2	[14.65, 71.8]	44.6	[15.9, 73.2]	74.1	[45.56, 102.7]	26.5	[−2.13, 55.0]	50.0	[21.37, 78.5]	non 0	0.3312
C18:1cis-9	609.0	[345, 873]	568.0	[304, 832]	649.0	[385, 912]	861.0	[597, 1125]	393.0	[129, 657]	614.0	[350, 878]	non 0	0.2714
C18:2*n* − 6	434.0	[267, 601]	407.0	[240, 574]	448.0	[281, 615]	576.0	[409, 742]	283.0	[116, 450]	411.0	[244, 578]	non 0	0.2889
C18:3*n* − 3	31.20	[10.3, 52.0]	41.80	[21.0, 62.7]	51.20	[30.3, 72.1]	67.30	[46.4, 88.1]	33.5	[12.7, 54.4]	52.50	[31.6, 73.4]	0	0.1353
C20:4*n* − 6	89.80	[83.5, 96.1]	84.60	[78.3, 90.8]	91.20	[84.9, 97.5]	89.60	[83.4, 95.9]	88.30	[82.0, 94.6]	93.30	[87.0, 99.6]	0	0.4745
C20:5*n* − 3	1.11 ^a^	[0.79, 1.42]	1.79 ^b^	[1.47, 2.10]	2.43 ^c^	[2.12, 2.74]	2.36 ^c^	[2.04, 2.67]	2.18 ^bc^	[1.86, 2.49]	2.30 ^c^	[1.99, 2.61]	0	<0.0001
C22:5*n* − 3	5.73	[3.77, 7.69]	7.78	[5.82, 9.74]	7.74	[5.77, 9.70]	9.94	[7.98, 11.90]	8.79	[6.83, 10.75]	8.32	[6.36, 10.28]	0	0.0758
C22:6*n* − 3	27.0 ^a^	[22.9, 31.1]	33.40 ^b^	[29.3, 37.5]	40.80 ^c^	[36.7, 44.9]	37.40 ^bc^	[33.3, 41.5]	39.80 ^c^	[35.7, 43.9]	45.50 ^c^	[41.4, 49.6]	non 0	<0.0001
^2^ ∑SFA	636.0	[434, 838]	602.0	[400, 804]	671.0	[469, 873]	820.0	[618, 1022]	467.0	[266, 669]	632.0	[430, 834]	non 0	0.2844
^3^ ∑MUFA	712.0	[405, 1019]	664.0	[356, 971]	751.0	[444, 1058]	1006.0	[699, 1313]	462.0	[154, 769]	722.0	[415, 1029]	non 0	0.2732
^4^ ∑ PUFA	624.0	[434, 814]	605.0	[415, 794]	670.0	[481, 860]	815.0	[626, 1005]	481.0	[291, 670]	641.0	[451, 831	non 0	0.2709
∑ *n* − 3 PUFA	65.90 ^a^	[44.0, 87.8]	86.0 ^ab^	[64.1, 107.9]	103.70 ^bc^	[81.8, 125.6]	118.80 ^c^	[96.9, 140.7]	85.50 ^ab^	[63.6, 107.4]	110.10 ^b^	[88.2, 132.0]	0	0.0103
∑ *n* − 6 PUFA	557.0	[385, 728]	517.0	[346, 689]	565.0	[394, 737]	695.0	[523, 867]	394.0	[222, 566]	529.0	[358, 701]	0	0.2845
Fat (%)	1.97	[1.27, 2.67]	1.87.0	[1.17, 2.57]	2.09	[1.39, 2.79]	2.64	[1.94, 3.34]	1.41	[0.71, 2.11]	2.00	[1.29, 2.69]	non 0	0.2719

Estimated marginal means (emmeans) with 95% upper confidence (UCL) and lower confidence limits (LCL); emmeans within a row with ^abc^ different superscript letters are significantly different at *p* < 0.05. ^1^ NC: 0 g flaxseed + no antioxidant sources, FS: 75 g flaxseed + no antioxidant sources, VE8: 75 g flaxseed + 800 mg α-tocopherol/kg, DA8: 75 g flaxseed + 800 mg *D. angustifolia* extract/kg, TS8: 75 g flaxseed + 800 mg *T. schimperi*/kg, CD8: 75 g flaxseed + 800 mg *C. domestica*/kg diet; ^2^ Sum of saturated fatty acids; ^3^ Sum of monounsaturated fatty acids; ^4^ Sum of polyunsaturated fatty acids.

**Table 5:** Effect of supplementing plant polyphenol extracts and flaxseed on lipid health indices and oxidative stability in raw and cooked breast meat of Sasso chickens.

Indices	Sample Type	^1^ Treatments												*p*-Value
		NC		FS		VE8		DA8		TS8		CD8		
		Mean	95% [LCL, UCL]	Mean	95% [LCL, UCL]	Mean	95% [LCL, UCL]	Mean	95% [LCL, UCL]	Mean	95% [LCL, UCL]	Mean	95% [LCL, UCL]	
*n* − 6/*n* − 3 ratio	raw	8.30 ^a^	[7.60, 8.99]	5.81 ^b^	[5.11, 6.51]	4.73 ^c^	[4.03, 5.43]	5.28 ^cb^	[4.58, 5.97]	4.65 ^c^	[3.95, 5.35]	4.50 ^c^	[3.80, 5.20]	<0.0001
	cooked	8.66 ^a^	[7.76, 9.57]	5.91 ^b^	[5.00, 6.81]	5.46 ^bc^	[4.55, 6.36]	5.63 ^b^	[4.72, 6.53]	4.60 ^c^	[ 3.69, 5.51]	4.81 ^bc^	[3.90, 5.71]	<0.0001
AI	raw	0.39	[0.38, 0.40]	0.39	[0.37, 0.39]	0.40	[0.39, 0.41]	0.39	[0.38, 0.40]	0.38	[0.37, 0.39]	0.39	[0.37, 0.40]	0.3870
	cooked	0.38	[0.36, 0.39]	0.37	[0.36, 0.38]	0.38	[0.36, 0.39]	0.37	[0.35, 0.38]	0.37	[0.36, 0.39]	0.37	[0.35, 0.38]	0.8385
TI	raw	0.77 ^a^	[0.74, 0.80]	0.70 ^b^	[0.67, 0.73]	0.70 ^b^	[0.67, 0.72]	0.70 ^b^	[0.66, 0.72]	0.67 ^b^	[0.64, 0.69]	0.66 ^b^	[0.63, 0.69]	<0.0001
	cooked	0.74 ^a^	[0.71, 0.77]	0.68 ^b^	[0.65, 0.71]	0.69 ^b^	[0.65, 0.71]	0.67 ^b^	[0.64, 0.70]	0.66 ^b^	[0.63, 0.69]	0.65 ^b^	[0.61, 0.67]	0.0002
h/H ratio	raw	2.42	[2.34, 2.50]	2.50	[2.42, 2.58]	2.41	[2.33, 2.50]	2.49	[2.41, 2.57]	2.49	[2.41, 2.57]	2.50	[2.42, 2.58]	0.4247
	cooked	2.56	[2.46, 2.66]	2.60	[2.50, 2.70]	2.59	[2.48, 2.69]	2.63	[2.53, 2.73]	2.57	[2.47, 2.68]	2.64	[2.54, 2.74]	0.8321
NVI	raw	1.56	[1.50, 1.62]	1.58	[1.52, 1.64]	1.58	[1.52, 1.64 ]	1.60	[1.54, 1.65]	1.57	[1.51, 1.63]	1.58	[1.53, 1.64]	0.9611
	cooked	1.73	[1.67, 1.79]	1.72	[1.66, 1.78]	1.75	[1.69, 1.81]	1.76	[1.70, 1.82]	1.77	[1.71, 1.83]	1.77	[1.71, 1.83]	0.4144
DFA (mg/100 g)	raw	949.0	[811, 1087]	939.0	[800, 1077]	1000.0	[862, 1139]	1014.0	[876, 1153]	853.0	[714, 991]	1031.0	[893, 1170]	0.4598
	cooked	1488.0	[957, 2019]	1414.0	[883, 1945]	1584.0	[1053, 2115]	2008.0	[1477, 2539]	1067.0	[536, 1598]	1514.0	[983, 2045]	0.2696
HFA (mg/100 g)	raw	306.0	[259, 352]	297.0	[251, 343]	328.0	[282, 375]	324.0	[278, 370]	269.0	[223, 315]	331.0	[285, 377}	0.3606
	cooked	469.0	[307, 631]	442.0	[280, 604]	492.0	[330, 654]	616.0	[454, 778]	331.0	[169, 493]	616.0	[454, 778]	0.2792
S/P	raw	0.51	[0.49, 0.52]	0.50	[0.48, 0.51]	0.51	[0.49, 0.52]	0.50	[0.48, 0.51]	0.50	[0.48, 0.51]	0.49	[0.47, 0.50]	0.6944
	cooked	0.47	[0.45, 0.48]	0.47	[0.45, 0.48]	0.47	[0.45, 0.49]	0.46	[0.44, 0.48]	0.49	[ 0.46, 0.51]	0.46	[0.43, 0.47]	0.3310
MDA (µg/g)	raw	2.55 ^a^	[2.05, 3.04]	2.60 ^a^	[2.10, 3.10]	2.00 ^ab^	[1.50, 2.49]	1.69 ^b^	[1.20, 2.19]	1.71 ^b^	[1.22, 2.21]	1.53 ^b^	[1.04, 2.03]	0.0121
	cooked	16.01 ^a^	[12.62, 19.4]	15.92 ^ab^	[12.53, 19.3]	16.44 ^a^	[13.05, 19.8]	11.17 ^bc^	[7.78, 14.6]	9.28 ^c^	[5.89, 12.7]	13.40 ^abc^	[10.01, 16.8)	0.0203

Estimated marginal means (emmeans) with 95% upper confidence (UCL) and lower confidence limits (LCL); emmeans within a row with ^abc^ different superscript letters are significantly different at *p* < 0.05. ^1^ NC: 0 g flaxseed + no antioxidant sources, FS: 75 g flaxseed + no antioxidant sources, VE8: 75 g flaxseed + 800 mg α -tocopherol/kg, DA8: 75 g flaxseed + 800 mg *D. angustifolia* extract/kg, TS8: 75 g flaxseed + 800 mg *T. schimperi*/kg, CD8: 75 g flaxseed + 800 mg *C. domestica*/kg diet, AI: atherogenic indices, TI: thrombogenic indices, h/H ratio: ratio of hypo/hyper cholestrolemic fatty acids, NVI: Nutritional value indices, DFA: Desirable fatty acid indices, HFA: hypercholestrolemic fatty acids, s/p: saturation indices.

## Data Availability

Data is contained within the article or Supplementary Material.

## Supplementary Materials

The following supporting information can be downloaded at: https://www.mdpi.com/article/10.3390/foods12010115/s1. Table S1: Ingredients (g/kg), nutrient levels (%), and fatty acid composition (%) of diets for dual-purpose Sasso T451A chickens. Table S2: Effect of supplementing plant polyphenol extracts and flaxseed on fat content and fatty acid concentration (mg/100 g) in raw breast muscle of Sasso T451A chickens. Table S3: Effect of supplementing plant polyphenol extracts and flaxseed on fat content and fatty acid concentration (mg/100 g) in cooked breast muscle of Sasso T451A chickens.
