# Supplementary material for: In Vitro Antioxidant Activities of Plant Polyphenol Extracts and Their Combined Effect with Flaxseed on Raw and Cooked Breast Muscle Fatty Acid Content, Lipid Health Indices and Oxidative Stability in Slow-Growing Sasso Chickens
Source: Foods. 2022 Dec 26;12(1):115. doi: 10.3390/foods12010115 (PMC9818817; doi:10.3390/foods12010115)
Supplement: Supplementary file 1 [file foods-12-00115-s001.zip › foods-2059754-supplementary.pdf]

Table S1. Ingredients, nutrient levels and fatty acid composition of diets for Sasso T451A chickens

Ingredients (g/kg)	Diets[1]						Flaxseed
	NC	FS	VE8	DA8	TS8	CD8	
Maize	468	405	405	405	405	405	
Soybean meal	60	55	55	55	55	55	
Flaxseed	0	75	75	75	75	75	
Wheat	148	140	140	140	140	140	
Sunflower	30	5	5	5	5	5	
Wheat bran	140	190	190	190	190	190	
Soybean full fat	35	10	10	10	10	10	
Meat and Bone	12	10	10	10	10	10	
Niger seed cake	34	37	37	37	37	37	
Salt	1	1	1	1	1	1	
Limestone	60	60	60	60	60	60	
Premix general*	10	10	10	10	10	10	
L-Lysine HCL	1	1	1	1	1	1	
DL-Methionine	1	1	1	1	1	1	
Vitamin E (mg/kg)	0	0	800	0	0	0	
D. angustifolia (mg/kg)	0	0	0	800	0	0	
T. schimperi (mg/kg)	0	0	0	0	800	0	
C. domestica (m/kg)	0	0	0	0	0	800	
Calculated composition							
Dry matter (%)	91.89	91.66	92.13	91.43	91.41	91.72	
ME (Kcal)	2999.62	2989.81	2997.12	2992.37	2989.91	2983.27	
Either Extract (%)	5.53	5.54	5.55	5.57	5.52	5.52	
Crude protein (%)	15.53	15.68	15.48	15.47	15.68	15.65	
Crude fibre (%)	5.52	5.44	5.27	5.46	5.56	5.47	
Calcium (%)	3.11	3.13	3.08	3.04	3.03	3.06	
Fatty acid composition (%)							
C14:0	0.17	0.14	0.12	0.12	0.13	0.14	0.06
C16:0	14.58	13.03	12.91	14.28	13.21	12.92	7.09
C18:0	3.43	4.29	4.19	3.48	3.91	4.27	4.63
C18:1*cis*-9	22.60	20.96	22.19	21.55	21.30	20.69	16.45
C18:2*n*-6	53.31	39.65	41.52	45.80	43.75	38.51	15.46
C18:3*n*-3	2.87	18.03	16.92	11.90	15.53	20.14	54.24
[2]SFA	19.58	18.82	18.74	19.38	18.75	18.68	12.48
[3]MUFA	24.27	22.55	23.61	23.13	22.78	22.20	17.44
[4]PUFA	56.15	58.63	57.65	57.49	58.47	59.12	70.08
n-3 PUFA	2.87	18.03	16.92	11.90	15.53	20.14	54.33
n-6 PUFA	53.35	39.69	41.55	45.85	43.79	38.55	15.76

n-6/n-3 ratio	19.08	2.10	2.58	3.94	2.98	1.87	0.29

[1]NC: 0 g flaxseed + no antioxidant sources, FS: 75 g flaxseed + no antioxidant sources, VE8: 75 g flaxseed + 800 mg α -tocopherol /kg, DA8: 75 g flaxseed + 800 mg *D. angustifolia* extract /kg ,TS8: 75 g flaxseed + 800 mg *T. schimperi* /kg, CD8: 75 g flaxseed + 800 mg *C. domestica* /kg diet, [2]Satutarted fatty acids; [3]Monounsaturated fatty acids; [4]polyunsaturated fatty acids.

*Mineral-vitamin mixtures provided per kg of feed: retinyl acetate (Vit A), 1.2 mg; cholecalciferol (Vit D) 0.00015 mg; all-rac-alpha-tocopherol acetate (E) 10 mg, menadione vitamin K3 0.8 mg; vitamin B1 0.6 mg; vitamin B2 (riboflavin) 2 mg; vitamin B3 (calcium-D-pantothenate) 3.6 mg; vitamin B6 2 mg; vitamin B12 (Cyanocobalamin) 0.01 mg; vitamin PP (Nicotinic acid) 12 mg; folic acid 0.4 mg; biotin 0.04 mg; Choline 259.5 mg; iron 18 mg; copper 6 mg; manganese 30 mg; zinc oxide 28 mg; iodine 0.8 mg; selenium 0.16 mg; calcium 492.6 mg, magnesium 5.07 mg; sodium 0.38, chloride 741.25 mg (Intraco Ltd. Jordaenskaai 24 2000 Antwerpen, Belgium).

Table S2. Effect of supplementing plant polyphenol extracts and flaxseed on fat content and fatty acid concentration (mg/100g) in raw breast muscle of Sasso chickens

Fatty acids (mg /100g)	Treatments												Random effect	p-value
	NC		FS		VE8		DA_8		TS_8		CD_8			
	Mean	95% [LCL, UCL]	Mean	95% [LCL, UCL]	Mean	95% [LCL, UCL]	Mean	95% [LCL, UCL]	Mean	95% [LCL, UCL]	Mean	95% [LCL, UCL]		
C10:0	0.75	[0.64, 0.84]	0.74	[0.64, 0.84]	0.76	[0.66, 0.86]	0.78	[0.68, 0.88]	0.74	[0.64, 0.84]	0.83	[0.73, 0.93]	0	0.7761
C12:0	0.84	[0.73, 0.95]	0.85	[0.74, 0.96]	0.95	[0.84, 1.05]	0.89	[0.79, 1.01]	0.78	[0.66, 0.88]	0.98	[0.86, 1.08]	non 0	0.1220
C14:0	7.41	[5.87, 8.94]	7.43	[5.90, 8.97]	8.37	[6.84, 9.90]	8.17	[6.64, 9.71]	6.37	[4.84, 7.90]	8.29	[6.76, 9.82]	0	0.3971
C15:0	1.42	[1.18, 1.66]	1.42	[1.18, 1.66]	1.34	[1.10, 1.58]	1.48	[1.24, 1.72]	1.24	[0.99, 1.48]	1.43	[1.19, 1.67]	0	0.7541
C16:0	297	[253, 342]	288	[244, 333]	319	[275, 364]	315	[270, 359]	262	[217, 306]	322	[277, 366]	0	0.3610
C17:0	2.29	[1.92, 2.67]	2.34	[1.97, 2.71]	2.34	[1.9, 2.72]	2.42	[2.05, 2.80]	2.01	[1.64, 2.38]	2.44	[2.07, 2.81]	0	0.6024
C18:0	114	[102.2, 126]	112	[99.7, 124]	119	[107.3, 131]	120	[108.3, 132]	101	[88.7, 113]	120	[107.9, 132]	0	0.1634
C20:0	0.98	[0.82, 1.13]	0.99	[0.84, 1.15]	0.96	[0.80, 1.12]	1.13	[0.97, 1.29]	0.81	[0.66, 0.97]	1.04	[0.88, 1.19]	0	0.1199
C21:0	0.28	[0.25, 0.31]	0.33	[0.29, 0.36]	0.31	[0.27, 0.33]	0.30	[0.27, 0.34]	0.29	[0.25, 0.32]	0.30	[0.27, 0.33]	0	0.4388
C22:0	1.46	[1.32, 1.60]	1.40	[1.26, 1.54]	1.29	[1.16, 1.43]	1.44	[1.30, 1.58]	1.35	[1.21, 1.48]	1.46	[1.32, 1.60]	non 0	0.4390
C23:0	1.44	[1.21, 1.67]	1.57	[1.34, 1.80]	1.48	[1.25, 1.71]	1.63	[1.40, 1.86]	1.62	[1.39, 1.85]	1.68	[1.46, 1.91]	non 0	0.6294
C24:0	0.79	[0.64, 0.93]	0.82	[0.68, 0.96]	0.67	[0.53, 0.81]	0.83	[0.68, 0.97]	0.76	[0.61, 0.90]	0.76	[0.61, 0.90]	non-0	0.6595
∑SFA	430	[372, 489]	420	[361, 478]	458	[399, 517]	455	[397, 514]	381	[323, 440]	462	[403, 521]	0	0.3362
C14:1cis-9	0.45	[0.29, 0.60]	0.41	[0.25, 0.56]	0.57	[0.42, 0.73]	0.51	[0.35, 0.66]	0.41	[0.25, 0.56]	0.57	[0.41, 0.72]	0	0.4426
C16:1cis-9	20.8	[15.2, 26.5]	19.5	[13.9, 25.2]	26.1	[20.4, 31.7]	22.8	[17.2, 28.5]	19.0	[13.3, 24.6]	25.6	[20.0, 31.3]	0	0.3114
C17:1cis-9	6.03	[5.43, 6.64]	5.70	[5.09, 6.30]	5.72	[5.12, 6.32]	5.50	[4.90, 6.11]	6.49	[5.89, 7.09]	6.99	[6.39, 7.59]	0	0.0056
C18:1trans-9	0.77	[0.50, 1.03]	0.61	[0.34, 0.87]	0.75	[0.48, 1.01]	0.71	[0.44, 0.97]	0.61	[0.34, 0.87]	0.78	[0.51, 1.04]	non 0	0.8715
C18:1trans-11	0.08	[-0.03, 0.20]	0.24	[0.12, 0.35]	0.18	[0.05, 0.29]	0.16	[0.04, 0.27]	0.14	[0.02, 0.26]	0.07	[-0.04, 0.18]	0	0.3571
C18:1cis-9	326	[262, 390]	322	[258, 386]	358	[294, 422]	357	[293, 421]	287	[223, 351]	363	[299, 427]	0	0.4900
C18:1cis-11	28.2	[24.3, 32.1]	26.5	[22.6, 30.4]	28.9	[25.1, 32.8]	27.8	[23.9, 31.7]	25.6	[21.7, 29.5]	28.8	[24.9, 32.7]	0	0.7839
C20:1cis-11	2.77	[2.19, 3.34]	2.83	[2.25, 3.40]	2.78	[2.21, 3.36]	3.15	[2.58, 3.73]	2.33	[1.75, 2.90]	2.95	[2.37, 3.52]	0	0.4630
C22:1cis-13	0.57	[0.43, 0.70]	0.56	[0.42, 0.69]	0.52	[0.38, 0.65]	0.60	[0.46, 0.73]	0.41	[0.27, 0.54]	0.58	[0.44, 0.71]	0	0.3690
∑MUFA	386	[311, 460]	378	[304 , 452]	424	[349, 498]	418	[344, 493]	342	[268, 416]	430	[356, 504]	0	0.5033
C18:2*n*-6	279	[232, 327]	271	[224, 319]	266	[218, 313]	291	[244, 339]	231	[184, 279]	282	[234, 329]	0	0.5525
C18:3*n*-6	1.44^{ab}	[1.11, 1.76]	1.55^{a}	[1.23, 1.87]	1.71^{a}	[1.39, 2.03]	1.66^{b}	[1.34, 1.97]	1.02^{b}	[0.69, 1.34]	1.65^{a}	[1.33, 1.97]	0	0.0267
C18:3*n*-3	16.9^{a}	[11.1, 22.6]	20.8^{ab}	[15.1, 26.6]	26.6^{bc}	[20.9, 32.4]	27.9^{bc}	[22.2, 33.7]	22.7^{abc}	[16.9, 28.4]	30.5^{c}	[24.8, 36.3]	0	0.0189
C18:2cis-9, trans 11	0.40	[0.32, 0.48]	0.39	[0.31, 0.46]	0.42	[0.34, 0.49]	0.49	[0.41, 0.57]	0.41	[0.33, 0.49]	0.45	[0.37, 0.53]	0	0.4301
C18:4n-3	0.24^{a}	[0.17, 0.30]	0.31^{ab}	[0.24, 0.38]	0.40^{b}	[0.33, 0.47]	0.37^{ab}	[0.30, 0.44]	0.25^{a}	[0.18, 0.32]	0.44^{c}	[0.36, 0.50]	0	0.0001
C20:2n-6	2.99	[2.66, 3.32]	2.85	[2.52, 3.18]	2.54	[2.21, 2.87]	2.87	[2.54, 3.20]	2.59	[2.26, 2.92]	2.87	[2.54, 3.20]	non 0	0.3196

C20:3*n*-9	0.72	[0.62, 0.82]	0.68	[0.57, 0.78]	0.72	[0.62, 0.82]	0.78	[0.68, 0.88]	0.70	[0.59, 0.80]	0.75	[0.64, 0.85]	0	0.7569
C20:3n-6	5.49	[4.95, 6.04]	5.21	[4.67, 5.75]	5.34	[4.80, 5.88]	5.67	[5.12, 6.21]	5.39	[4.85, 5.93]	5.76	[5.22, 6.31]	non 0	0.6931
C20:3n-3	0.35^{a}	[0.24, 0.45]	0.46^{ac}	[0.35, 0.56]	0.57^{b}	[0.46, 0.67]	0.68^{b}	[0.57, 0.78]	0.59^{bc}	[0.48, 0.70]	0.67^{b}	[0.56, 0.77]	non 0	<0.0001
C20:4*n*-6	84.20	[78.1, 90.4]	83.2	[77.0, 89.4]	86.5	[80.3, 92.7]	82.5	[76.3, 88.7]	79.9	[73.7, 86.0]	86.6	[80.4, 92.7]	non 0	0.6225
C20:5*n*-3	0.31^{a}	[0.003, 0.62]	0.66^{a}	[0.35, 0.97]	1.36^{b}	[1.05, 1.67]	1.17^{b}	[0.86, 1.47]	1.15^{b}	[0.84, 1.46]	1.49^{b}	[1.17, 1.79]	non 0	<0.0001
C22:4n-6	13.86^{a}	[12.31, 15.4]	9.92^{b}	[8.38, 11.5]	8.90^{b}	[7.36, 10.4]	9.52^{b}	[7.98, 11.1]	9.42^{b}	[7.88, 11.0]	9.17^{b}	[7.62, 10.7]	non 0	0.0004
C22:5*n*-6	10.76^{a}	[9.07, 12.45]	7.31^{b}	[5.62, 8.99]	5.73^{b}	[4.04, 7.42]	5.62^{b}	[3.93, 7.31]	5.40^{b}	[3.71, 7.09]	5.58^{b}	[3.89, 7.27]	non 0	0.0003
C22:5*n*-3	8.26^{a}	[7.36, 9.15]	9.26^{ac}	[8.37, 10.15]	11.40^{b}	[10.51, 12.29]	10.42^{bc}	[9.52, 11.31]	10.59^{b}	[9.70, 1.49]	10.99^{b}	[10.1,11.88]	0	<0.0001
C22:6*n*-3	23.8^{a}	[21.1, 26.6]	34.7^{b}	[31.9, 37.5]	39.6^{c}	[36.8, 42.3]	34.6^{bd}	[31.8, 37.4]	36.9^{bcd}	[34.1, 39.7]	43.3^{c}	[40.5, 46.0]	non 0	<0.0001
∑ PUFA	449	[395, 504]	448	[394, 503]	458	[403, 512]	475	[421, 530]	408	[354, 463]	482	[427, 536]	0	0.4594
∑ *n*-3 PUFA	49.9^{a}	[43.3, 56.6]	66.2^{b}	[59.6, 72.9]	79.9^{bc}	[73.3, 86.6]	75.2^{bc}	[68.5, 81.8]	72.2^{bc}	[65.5, 78.8]	87.3^{c}	[80.7, 94.0]	non 0	<0.0001
∑ *n*-6 PUFA	398	[348, 449]	381	[331, 432]	376	[326, 427]	399	[348, 450]	335	[285, 386]	393	[343, 444]	0	0.4642
Fat (%)	1.27	[1.09, 1.46]	1.25	[1.06, 1.44]	1.34	[1.15, 1.52]	1.35	[1.16, 1.53]	1.12	[0.93, 1.31]	1.37	[1.18, 1.55]	0	0.4040

Estimated marginal means (emmeans) with 95% upper confidence (UCL) and lower confidence limits (LCL); Emmeans within a row with different superscript letters are significantly different at p≤0.05).Treatments: NC: 0 g flaxseed + no antioxidant sources, FS: 75 g flaxseed + no antioxidant sources, VE8: 75 g flaxseed + 800 mg α -tocopherol /kg, DA8: 75 g flaxseed + 800 mg *D. angustifolia* extract /kg ,TS8: 75 g flaxseed + 800 mg *T. schimperi* /kg, CD8: 75 g flaxseed + 800 mg *C. domestica* /kg diet, SFA: Saturated fatty acids (C10:0 + C12:0 + C14:0 + C15:0 + C16:0 + C17:0 + C18:0+ C20:0 + C21:0 + C22:0 + C23:0 + C24:0 + C26:0); MUFA: Monounsaturated fatty acids (C14:1cis-9 + C16:1cis-9 + C17:1cis-9 + C18:1trans-9 + C18:1trans-11 + C18:1cis-9 + C18:1cis-11 + C20:1cis-11 + C22:1cis-13); PUFA: Polyunsaturated fatty acids (C18:2n-6 + C18:3n-6 + C18:3n-3 + C18:2cis-9,trans-11 + C18:4n-3 + C20:2n-6 + C20:3 n-9 + C20:3n-6 + C20:3n-3 + C20:4n-6 + C20:5n-3 + C22:4n-6 + C22:5n-6 + C22:5n-3 + C22:6n-3), . ∑ *n*-3 PUFA: Sum n-3 PUFA (C18:3n-3 + C18:4n-3 + C20:3n-3 + C20:5n-3 + C22:5n-3 + C22:6n-3); ∑ *n*-6 PUFA : Sum of n-3 PUFA (C18:2n-6 +C18:3n-6 + C20:2n-6 + C20:3n-6 + C20:4n-6 + C22:4n-6 + C22:5n-6).

Table S3. Effect of supplementing plant polyphenol extracts and flaxseed on fat content and fatty acid concentration (mg/100g) in cooked breast muscle of Sasso chickens

Fatty acids (mg /100g)	Treatments													
	NC		FS		VE8		DA8		TS8		CD8		Random effect	p-value
	Mean	95% [LCL, UCL]	Mean	95% [LCL, UCL]	Mean	95% [LCL, UCL]	Mean	95% [LCL, UCL]	Mea n	95% [LCL,UCL]	Mean	95% [LCL, UCL]		
C10:0	0.63	[0.40, 0.84]	0.89	[0.67, 1.11]	0.79	[0.56, 1.00]	0.99	[0.77, 1.21]	0.82	[0.59, 1.04]	0.89	[0.67, 1.11]	non 0	0.2714
C12:0	1.21	[0.92, 1.50]	1.15	[0.86, 1.43]	1.26	[0.97, 1.55]	1.47	[1.18, 1.76]	0.90	[0.61, 1.19]	1.23	[0.94, 1.52]	non 0	0.1572
C14:0	12.15^{a}	[7.68,16.6]	11.16^{ab}	[6.69, 15.6]	12.71^{a}	[8.24, 17.2]	15.99^{ab}	[11.52, 20.5]	7.98^{b}	[3.51, 12.4]	11.53^{ab}	[7.06, 16.0]	non 0	0.2562
C15:0	2.23	[1.49, 2.96]	2.16	[1.43, 2.90]	2.27	[1.53, 3.00]	2.87	[2.13, 3.60]	1.59	[0.85, 2.32]	2.10	[1.37, 2.84]	non 0	0.2960
C16:0	456	[298, 613]	430	[273, 587]	478	[321, 635]	599	[441, 756]	322	[165, 479]	453	[296, 610]	non 0	0.2803
C17:0	3.62	[2.38, 4.85	3.52	[2.29, 4.76]	3.91	[2.68, 5.15]	4.75	[3.52, 5.98]	2.62	[1.38, 3.85]	3.41	[2.17, 4.64]	non 0	0.2849
C18:0	152	[115.3, 189]	145	[108.4,182]	163	[125.9, 200]	187	[149.8, 224]	125	[87.6, 162]	151	[114.3, 188]	non 0	0.3012
C20:0	1.74	[1.18, 2.29]	1.69	[1.13, 2.24]	1.82	[1.27, 2.37]	2.32	[1.76, 2.87]	1.42	[0.86, 1.97]	1.75	[1.20, 2.30]	non 0	0.3406
C21:0	0.33	[0.24, 0.41]	0.35	[0.26, 0.43]	0.34	[0.25, 0.42]	0.43	[0.34, 0.52]	0.34	[0.26, 0.43]	0.38	[0.29, 0.47]	0	0.4838
C22:0	1.11	[0.94, 1.27]	1.12	[0.95, 1.28]	1.27	[1.10, 1.43]	1.34	[1.17, 1.50]	1.08	[0.91, 1.24]	1.24	[1.07, 1.40]	0	0.1431
C23:0	0.74	[0.34, 1.14]	0.69	[0.29, 1.09]	0.72	[0.31, 1.12]	1.02	[0.62, 1.42]	0.75	[0.35, 1.15]	0.88	[0.48, 1.28]	non 0	0.8385
C24:0	1.36	[1.20, 1.51]	1.27	[1.12, 1.43]	1.38	[1.22, 1.53]	1.42	[1.27, 1.57]	1.25	[1.10, 1.40]	1.38	[1.23, 1.53]	0	0.5396
∑SFA	636	[434, 838]	602	[400, 804]	671	[469, 873]	820	[618, 1022]	467	[266, 669]	632	[430, 834]	non 0	0.2844
C14:1cis-9	1.10	[0.40, 1.80]	1.06	[0.36, 1.75]	1.00	[0.30, 1.70]	1.74	[1.04, 2.43]	0.58	[-0.11, 1.28]	1.15	[0.45, 1.85]	non 0	0.3424
C16:1cis-9	46.5	[17.93, 75.1]	43.2	[14.65, 71.8]	44.6	[15.9, 73.2]	74.1	[45.56, 102.7]	26.5	[-2.13, 55.0]	50.0	[21.37, 78.5]	non 0	0.3312
C17:1cis-9	7.51	[6.54, 8.47]	7.46	[6.49, 8.42]	7.86	[6.90, 8.83]	8.38	[7.42, 9.34]	7.55	[6.59, 8.52]	9.15	[8.19, 10.12]	0	0.0891
C18:1trans-9	1.73	[1.35, 2.09]	1.45	[1.08, 1.82]	1.72	[1.34, 2.09]	1.54	[1.16, 1.90]	1.13	[0.75, 1.49]	1.50	[1.13, 1.87]	non 0	0.2276
C18:1trans-11	0.46	[-0.203, 1.13]	0.65^{a}	[-0.015, 1.32]	0.37^{ab}	[-0.29, 1.048]	1.27	[0.60, 1.93]	0.28	[-0.38, 0.95]	0.26	[-0.40, 0.93]	0	0.2649
C18:1cis-9	609	[345, 873]	568	[304, 832]	649	[385, 912]	861	[597, 1125]	393	[129, 657]	614	[350, 878]	non 0	0.2714
C18:1cis-11	39.4	[27.6, 51.3]	36.3	[24.4, 48.2]	40.3	[28.4, 52.2]	49.4	[37.5, 61.3]	28.3	[16.4, 40.1]	39.9	[28.0, 51.8]	non 0	0.2622
C20:1cis-11	4.93	[2.96, 6.90]	4.45	[2.48, 6.42]	5.27	[3.29, 7.24]	6.89	[4.92, 8.86]	3.36	[1.39, 5.34]	4.62	[2.65, 6.59]	non 0	0.2390
C22:1cis-13	1.28	[0.84, 1.71]	1.16	[0.72, 1.59]	1.19	[0.76, 1.63]	1.70	[1.26, 2.13]	0.82	[0.39, 1.26]	1.22	[0.78, 1.65]	non 0	0.1523
∑MUFA	712	[405, 1019]	664	[356, 971]	751	[444, 1058]	1006	[699, 1313]	462	[154, 769]	722	[415, 1029]	non 0	0.2732
C18:2*n*-6	434	[267, 601]	407	[240,574]	448	[281,615]	576	[409,742]	283	[116,450]	411	[244,578]	non 0	0.2889
C18:3*n*-6	2.60	[1.25, 3.95]	2.71	[1.35, 4.05]	3.03	[1.68, 4.38]	3.89	[2.53, 5.24]	1.82	[0.46, 3.17]	2.49	[1.14, 3.84]	non 0	0.4028
C18:3*n*-3	31.2	[10.3, 52.0]	41.8	[21.0, 62.7]	51.2	[30.3, 72.1]	67.3	[46.4, 88.1]	33.5	[12.7, 54.4]	52.5	[31.6, 73.4]	0	0.1353
C18:2cis-9,	0.61	[0.39, 0.79]	0.61	[0.41, 0.81]	0.56	[0.36, 0.76]	0.74	[0.54, 0.93]	0.47	[0.26, 0.66]	0.65	[0.44, 0.84]	0	0.5204

tran-11														
C18:4n-3	0.47^{a}	[0.15, 0.79]	0.61	[0.28, 0.93]	0.75	[0.42, 1.06]	0.98	[0.66, 1.31]	0.54	[0.21, 0.85]	0.76	[0.44, 1.09]	0	0.2393
C20:2n-6	3.64	[3.01, 4.27]	3.28	[2.65, 3.91]	3.46	[2.83, 4.09]	3.87	[3.24, 4.50]	2.84	[2.21, 3.47]	3.35	[2.72, 3.98]	non 0	0.2987
C20:3*n*-9	0.70	[0.60, 0.79]	0.71	[0.62, 0.81]	0.73	[0.63, 0.82]	0.83	[0.73, 0.92]	0.70	[0.60, 0.79]	0.80	[0.70, 0.89]	0	0.2399
C20:3n-6	5.41	[4.73, 6.09]	5.81	[5.13, 6.49]	5.95	[5.27, 6.63]	6.33	[5.65, 7.01]	5.16	[4.48, 5.84]	5.78	[5.10, 6.46]	0	0.1934
C20:3n-3	0.44	[0.30, 0.57]	0.64	[0.49, 0.77]	0.71	[0.57, 0.85]	0.81	[0.67, 0.95]	0.67	[0.53, 0.81]	0.75	[0.60, 0.88]	0	0.006*
C20:4*n*-6	89.8	[83.5, 96.1]	84.6	[78.3, 90.8]	91.2	[84.9, 97.5]	89.6	[83.4, 95.9]	88.3	[82.0, 94.6]	93.3	[87.0, 99.6]	0	0.4745
C20:5*n*-3	1.11^{a}	[0.79, 1.42]	1.79^{b}	[1.47, 2.10]	2.43^{c}	[2.12, 2.74]	2.36^{c}	[2.04, 2.67]	2.18^{bc}	[1.86, 2.49]	2.30^{c}	[1.99, 2.61]	0	<0.0001
C22:4n-6	11.57^{a}	[10.38, 12.75]	8.93^{b}	[7.74, 10.11]	8.29^{b}	[7.11, 9.47]	9.27^{b}	[8.08, 10.45]	7.98^{b}	[6.80, 9.17]	8.06^{b}	[6.87, 9.24]	0	<0.0001
C22:5*n*-6	9.96^{a}	[8.53, 11.38]	5.20^{b}	[3.78, 6.62]	5.53^{b}	[4.11, 6.95]	6.31^{b}	[4.89, 7.73]	4.43^{b}	[3.01, 5.85]	5.22^{b}	[3.80, 6.64]	0	<0.0001
C22:5*n*-3	5.73	[3.77, 7.69]	7.78	[5.82, 9.74]	7.74	[5.77, 9.70]	9.94	[7.98, 11.90]	8.79	[6.83, 10.75]	8.32	[6.36, 10.28]	0	0.0758
C22:6*n*-3	27.0^{a}	[22.9, 31.1]	33.4^{b}	[29.3, 37.5]	40.8^{c}	[36.7, 44.9]	37.4^{bc}	[33.3, 41.5]	39.8^{c}	[35.7, 43.9]	45.5^{c}	[41.4, 49.6]	non 0	<0.0001
∑ PUFA	624	[434, 814]	605	[415, 794]	670	[481, 860]	815	[626, 1005]	481	[291, 670]	641	[451, 831	non 0	0.2709
∑ *n*-3 PUFA	65.9^{a}	[44.0, 87.8]	86.0^{ab}	[64.1, 107.9]	103.7^{bc}	[81.8, 125.6]	118.8^{c}	[96.9, 140.7]	85.5^{ab}	[63.6, 107.4]	110.1^{b}	[88.2, 132.0]	0	0.0103
∑ *n*-6 PUFA	557	[385, 728]	517	[346, 689]	565	[394, 737]	695	[523, 867]	394	[222 , 566]	529	[358, 701]	0	0.2845
Fat (%)	1.97	[1.27, 2.67]	1.87	[1.17, 2.57]	2.09	[1.39, 2.79]	2.64	[1.94, 3.34]	1.41	[0.71, 2.11]	2.00	[1.29, 2.69]	non 0	0.2719

Estimated marginal means (emmeans) with 95% upper confidence (UCL) and lower confidence limits (LCL); Emmeans within a row with different superscript letters are significantly different at $p \leq 0.05$).Treatments: NC: 0 g flaxseed + no antioxidant sources, FS: 75 g flaxseed + no antioxidant sources, VE8: 75 g flaxseed + 800 mg α -tocopherol /kg, DA8: 75 g flaxseed + 800 mg *D. angustifolia* extract /kg ,TS8: 75 g flaxseed + 800 mg *T. schimperi* /kg, CD8: 75 g flaxseed + 800 mg *C. domestica* /kg diet, SFA: Saturated fatty acids (C10:0 + C12:0 + C14:0 + C15:0 + C16:0 + C17:0 + C18:0+ C20:0 + C21:0 + C22:0 + C23:0 + C24:0 + C26:0); MUFA: Monounsaturated fatty acids (C14:1cis-9 + C16:1cis-9 + C17:1cis-9 + C18:1trans-9 + C18:1trans-11 + C18:1cis-9 + C18:1cis-11 + C20:1cis-11 + C22:1cis-13); PUFA: Polyunsaturated fatty acids (C18:2n-6 + C18:3n-6 + C18:3n-3 + C18:2cis-9,trans-11 + C18:4n-3 + C20:2n-6 + C20:3 n-9 + C20:3n-6 + C20:3n-3 + C20:4n-6 + C20:5n-3 + C22:4n-6 + C22:5n-6 + C22:5n-3 + C22:6n-3), . ∑ *n*-3 PUFA: Sum n-3 PUFA (C18:3n-3 + C18:4n-3 + C20:3n-3 + C20:5n-3 + C22:5n-3 + C22:6n-3); ∑ *n*-6 PUFA : Sum of n-3 PUFA (C18:2n-6 +C18:3n-6 + C20:2n-6 + C20:3n-6 + C20:4n-6 + C22:4n-6 + C22:5n-6).
